# Associations between an international COVID-19 job exposure matrix and SARS-CoV-2 infection among 2 million workers in Denmark

**DOI:** 10.5271/sjweh.4099

**Published:** 2023-09-01

**Authors:** Sophie van der Feltz, Vivi Schlünssen, Ioannis Basinas, Luise M Begtrup, Alex Burdorf, Jens PE Bonde, Esben M Flachs, Susan Peters, Anjoeka Pronk, Zara A Stokholm, Martie van Tongeren, Karin van Veldhoven, Karen M Oude Hengel, Henrik A Kolstad

**Affiliations:** 1 Department of Occupational Medicine, Danish Ramazzini Center, Aarhus University Hospital, Aarhus N, Denmark.; 2 Department of Public Health, Research Unit for Environment, Occupation and Health, Danish Ramazzini Center, Aarhus University, Bartholins Allé 2, 8000 Aarhus C, Denmark.; 3 Center for Occupational and Environmental Health, Division of Population Health, Health Services Research and Primary Care, School of Health Sciences, Faculty of Biology, Medicine and Health, The University of Manchester, Manchester, UK.; 4 Department of Public Health, University of Copenhagen, København K, Denmark.; 5 Department of Occupational and Environmental Medicine, Copenhagen University Hospital - Bispebjerg and Frederiksberg, Copenhagen, Denmark; 6 Erasmus Medical Center Rotterdam, Department of Public Health, CN Rotterdam, The Netherlands.; 7 Institute for Risk Assessment Sciences, Utrecht University, CM Utrecht, The Netherlands.; 8 Netherlands Organisation for Applied Scientific Research TNO, Unit Healthy Living, BE Leiden, The Netherlands.; 9 London School of Hygiene & Tropical Medicine, Faculty of Epidemiology and Public Health, Department of Non-Communicable Disease Epidemiology, London, UK.; 10 Institute of Clinical Medicine, Aarhus University, Aarhus N, Denmark.

**Keywords:** coronavirus, infection waves, JEM, occupational exposure, SARS-Cov-2 exposure

## Abstract

**Objectives:**

This study investigates the associations between the Danish version of a job exposure matrix for COVID-19 (COVID-19-JEM) and Danish register-based SARS-CoV-2 infection information across three waves of the pandemic. The COVID-19-JEM consists of four dimensions on transmission: two on mitigation measures, and two on precarious work characteristics.

**Methods:**

The study comprised 2 021 309 persons from the Danish working population between 26 February 2020 and 15 December 2021. Logistic regression models were applied to assess the associations between the JEM dimensions and overall score and SARS-CoV-2 infection across three infection waves, with peaks in March–April 2020, December–January 2021, and February–March 2022. Sex, age, household income, country of birth, wave, residential region and during wave 3 vaccination status were accounted for.

**Results:**

Higher risk scores within the transmission and mitigation dimensions and the overall JEM score resulted in higher odds ratios (OR) of a SARS-CoV-2 infection. OR attenuated across the three waves with ranges of 1.08–5.09 in wave 1, 1.06–1.60 in wave 2, and 1.05–1.45 in those not (fully) vaccinated in wave 3. In wave 3, no associations were found for those fully vaccinated. In all waves, the two precarious work dimensions showed weaker or inversed associations.

**Conclusions:**

The COVID-19-JEM is a promising tool for assessing occupational exposure to SARS-CoV-2 and other airborne infectious agents that mainly spread between people who are in close contact with each other. However, its usefulness depends on applied restrictions and the vaccination status in the population of interest.

Since its appearance in 2019 (1, 2), the severe acute respiratory syndrome coronavirus 2 (SARS-CoV-2) and its associated coronavirus disease (COVID-19) spread rapidly around the world causing the World Health Organization (WHO) to declare a pandemic on 11 March 2020 (3). The COVID-19 pandemic held the world in its grip, forcing governments to take far-reaching measures such as social distancing, travel restrictions, large scale testing and later mass vaccination campaigns. The pandemic and its associated government measures impacted both the population as a whole and, in specific ways, the working population.

Several studies have shown that occupation played a role in infection risk (4–8) and that the workplace was one of the key settings to be infected with SARS-CoV-2 (9). High risks have been observed for essential workers such as medical support staff, social care workers, transport workers and workers in education (10–13). The risk of SARS-CoV-2 infection differed not only across occupations but also across time, with healthcare workers at the highest relative risk in the first period of the pandemic (6, 10, 13). Other studies indicate that occupation is associated with COVID-19 related mortality (14–16), where higher excess mortality rates were found in sectors of healthcare, food and agriculture, transportation and logistics, manufacturing and facilities (15, 17).

To estimate the exposure to SARS-CoV-2 across occupations, an international COVID-19 job exposure matrix (JEM) was developed (18). JEM are common and useful tools to estimate exposure to a potential occupational health hazard (19), especially when exposure data at the individual level is difficult or even impossible to obtain. Ten occupational epidemiologists from Denmark, The Netherlands and the United Kingdom (UK) established the COVID-19-JEM, which contains eight different dimensions of exposure affecting factors. All job titles within the International Standard Classification of Occupations from 2008 (ISCO-08) were attributed with a risk score on each of the eight dimensions separately. Risk scores varied between countries, and therefore risk scores within the COVID-19-JEM were separately presented for Denmark, The Netherlands and the UK. The Dutch and UK versions of the COVID-19-JEM have already been validated (20, 21) where results from six of the eight dimensions supported that higher risk scores were associated with higher infection rates.

The current study focuses on the Danish version of the COVID-19-JEM. The Danish Government and health authorities reacted to the pandemic rapidly by declaring a national lockdown in early March 2020 with the practical implications of, for example, closure of on-site education and daycare facilities, the urging of people with non-essential occupations to work from home, restriction of air travel and discouragement of the use of public transport. Similar but less strict measures were applied related to infection peaks in wave 2 and 3. The Danish healthcare system was relatively well-prepared and the high level of trust in the Government (22) was illustrated by high vaccination rates, with 83.2% of the total population being fully vaccinated (vaccinated with the last dose of primary series) in November 2022 (23). The occupational exposure probability may differ across periods amid the different governmental measures taken and the availability of vaccinations. Therefore, this study aimed to investigate the associations between the COVID-19-JEM and SARS-CoV-2 infections across three infection waves with infection peaks in March–April 2020, December–January 2021 and February–March 2022 (23) using national register data (24).

## Methods

### Data and population

**Figure 1:**
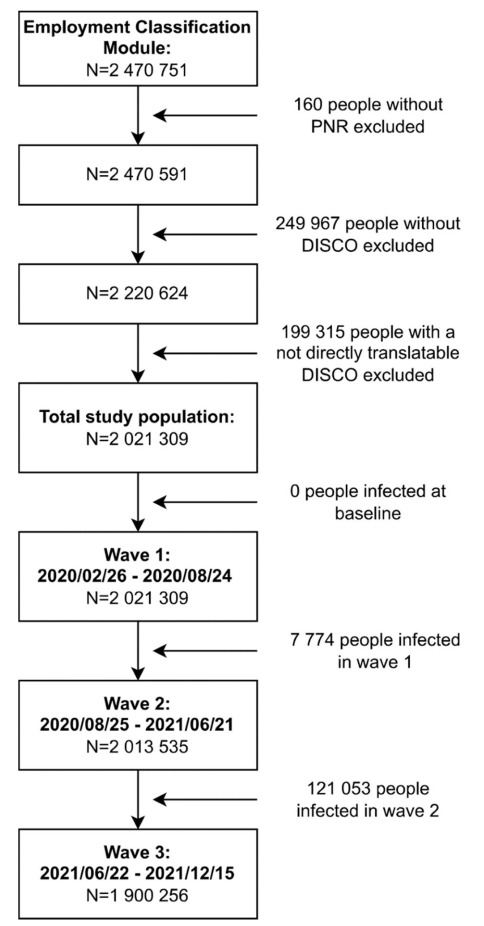
Flowchart of the study population. [PNR=personal idenitification number; DISCO= Danish version of the International Standard Classification of Occupations.]

Occupational and demographic data of the employed Danish population (aged 20–69 years) were retrieved from Statistics Denmark. Statistics Denmark also provided access to SARS-CoV-2 test data, which originated from the Danish Microbiology database (MiBa) hosted at the Danish Health Data Authority. On 11 January 2022, 2 470 751 persons were available for this study using the nationwide population register and an additional nationwide register with occupational data (Employment Classification Module) coupled with national SARS-CoV-2 test data and data on vaccination status. From the Employment Classification Module, we used the population’s most up-to-date DISCO [Danish version of the International Standard Classification of Occupations (ISCO-08)] codes retrieved on 31 December 2019. The last available national SARS-CoV-2 test data dated from 12 December 2021. Participants were excluded if their personal ID number was unknown (N=160), when they did not have information on occupation as recorded by the DISCO code (N=249 967) and when their DISCO code was not directly translatable to a four-digit ISCO-08 code (N=199 315), either because it was a specific Danish code or because they had a DISCO code on a higher, overarching level. The final study population consisted of 2 021 309 persons (figure 1).

To investigate the associations between the COVID-19-JEM and SARS-CoV-2 infection taking peak infection periods into consideration, three waves were defined by selecting the period between two dates with the lowest number of positive tests, covering the peak of infections in between. The first wave lasted from the first available COVID-19 test data on 26 February 2020 to 24 August 2020, the second wave from 25 August 2020 to 21 June 2021, and the third wave from 22 June 2021 to the last available test data on 12 December 2021. For each wave, we created a study population. The first wave included the total study population (N=2 021 309) since no one had tested positive at baseline. For the second wave (N=2 013 535), 7774 people were excluded because they had tested positive during wave 1. For the third wave (N=1 900 256), another 121 053 people were excluded because they had tested positive during wave 2.

### The COVID-19-JEM

The COVID-19-JEM was developed by ten occupational exposure and epidemiology experts who determined the dimensions of exposure to be included in the JEM and defined the risk scores per dimension (18). The COVID-19-JEM contains eight dimensions: four on transmission risk (number of contacts, nature of contacts, contaminated workspaces, and location), two on mitigation measures (social distancing and face covering), and two indicating precarious work (income insecurity and percentage of migrants) (18). For each dimension, all 436 occupations within the ISCO-08 were assigned an exposure probability (risk score) of 0–3 (no, low, intermediate, and high probability). For the current study, the Danish COVID-19-JEM was applied, of which all dimensions were based on expert assessments only (18). The dimensions and the implication of their risk scores are described in table 1.

**Table 1:** Dimensions of the COVID-19-JEM with the definitions and implications for their risk scores (Oude Hengel et al, 2022). [ 0=no risk; 1=low risk, 2=intermediate risk; 3= high risk.]

Dimension	Risk score	Definition risk score
Number of contacts (number of workers in close vicinity)	0	Homeworkers, or not working with others
	1	<10 contacts per day
	2	10–30 contacts per day
	3	>30 contacts per day
Nature of contacts (contacts with coworkers, general public or patients with COVID-19)	0	Homeworkers, or not working with others
	1	Contact with co-workers only
	2	Contact with general public
	3	Regular contacts with suspected or diagnosed COVID-19 patients
Contaminated workspaces (risk through contaminated work surfaces and materials)	0	Homeworkers, or not working with others
	1	Frequently (≥10 times a day)) sharing materials or surfaces with co-workers
	2	Sometimes (<10 times a day) sharing materials or surfaces with general public
	3	Frequently (≥10 times a day)) sharing materials or surfaces with general public
Work location (indoors or outdoors)	0	Homeworkers, or not working with others
	1	Working mostly outside
	2	Working partly inside (1-4 hours per day)
	3	Working mostly inside (>4 hours per day)
Social distance (the possibility to keep ≥ 1 meter of social distance)	0	Homeworkers, or not working with others
	1	Social distancing can always be maintained
	2	Social distancing cannot always be maintained
	3	Social distancing can never be maintained
Face covering (usage of face covering)	0	Homeworkers, or not working with others
	1	Wearing face covering at the worksite
	2	Wearing face covering during specific activities, but not always while in proximity of others
	3	Activities in proximity of others which cannot be done when wearing face covering
Income insecurity (proportion of workers with high income insecurity) ^a^	0	<1%
	1	1–10%
	2	11–25%
	3	>25%
Migrants (proportion of labour migrants)	0	<1%
	1	1–10%
	2	11–25%
	3	>25%

^a^ Due to the pandemic, eg, due to zero-hour contracts, casual work and day labour.

To match the Danish population with their assigned risk scores from the COVID-19-JEM, we used four-digit DISCO-08 codes. The COVID-19-JEM further divided some healthcare occupations into subgroups based upon industry (ISIC codes). For this study, we only investigated the overarching ISCO-08 codes. Therefore, the rounded means of the industry-specific risk scores were used to assign a risk score to their overarching ISCO-08 codes.

Finally, risk scores of all 8 dimensions were combined into a total sum score (overall JEM score) of 0–24, which was subsequently categorized in groups: 0, 1–8, 9–12, 13–16, 17–20, and 21–24. As the researchers who developed the COVID-19-JEM did not provide a group categorization, we categorized these groups based on no occupational risk (score group 0), low risk in all dimensions or only high risk in some dimensions (score group 1–8), and higher occupational risk (equally divided in four subsequent score groups).

### SARS-CoV-2 infection

Both polymerase chain reaction (PCR) and rapid antigen tests were provided free of charge to the Danish population at governmental test centers with no requirement of having symptoms or other indications for testing. Results of PCR tests and rapid antigen tests for SARS-CoV-2 were combined and dichotomized (positive/negative). When a person had at least one positive SARS-CoV-2 test (either PCR or rapid antigen), they were classified as infected, while persons with no or only negative tests were classified as not-infected.

### Test frequency

In each wave and over the total time period, average test frequency was investigated for the different risk scores of all dimensions. To gain inside in the average test frequency per person within these groups, the amount of tests was divided by the population of interest.

### Statistical methods

Characteristics of the study population were presented by absolute and relative frequencies. Logistic regression analyzes were performed to investigate the associations between the eight dimensions separately and the overall JEM score and SARS-CoV-2 infection. Analyzes were adjusted for sex, age, region, country of birth, and pre-tax household income. Sex (male/female) and pre-tax household income (<the average of 563 000 and ≥563 000 DKK (25) were included as dichotomous variables. Age was included as a categorical variable in five-year age groups. Regions were divided into capital, metropolitan, provincial, commuter, and rural as defined by Statistics Denmark (26). Country of origin was categorized as Denmark, European Union (EU), Western but not EU, and other.

As interaction between infection and wave was evident, associations were examined for both the total study period and the three waves separately. In wave 3, a significant interaction was found between infection and vaccination status, and the analyzes of wave 3 were therefore stratified by vaccination status. Vaccination status was only considered in wave 3 (fully or not (fully) vaccinated at the start of wave 3) as barely anyone was (fully) vaccinated in waves 1 and 2. Fully vaccinated implied either having received one Janssen vaccination or two vaccinations of AstraZeneca, Moderna or Pfizer-BioNTech. Finally, region was also identified as an effect modifier and additional analyzes were therefore stratified by region as a dichotomous variable (capital region versus other regions).

Analyzes were repeated using a test negative design (27). This meant that in each wave, people were excluded when they had not been tested during that wave. For the total study period it meant that to be included, people needed to have been tested at least once over the whole time span.

RStudio version 4.0.3 was used to conduct all statistical analyzes.

## Results

Table 2 gives an overview of the characteristics of the total study population across the overall JEM score. Of the 225 209 persons that were infected over the entire study period, 171 724 (76.3%) tested positive with a PCR test and 53 485 (23.7%) tested positive with an antigen test. Generally, a higher percentage of persons were infected by increasing overall COVID-19-JEM score, with a highly significant difference between score group 0 (10.5% infected) and score group 21–24 (16.2% infected), *Χ*^2^ (1)= 601.5, P<0.001. Sex distribution fluctuated across the different overall JEM score groups, with more males in score groups 1–12 and 21–24 and more females in score groups 0 and 13–20. The workers in metropolitan, provincial, and commuter regions were evenly distributed across all score groups. However, a larger share of people living in the capital region was found in score groups 0–8 and 21–24, while a larger share of people living in the rural region was found in score groups 9–20. Percentages of people born in Denmark decreased with increasing overall JEM score (from 90.6% in score group 0 to 75.9% in score groups 21–24). Percentages of people with a household income below average increased with overall JEM score (25.8% in score group 0 and 63.2% in score group 21–24). Characteristics of the population across the total time period and population outlined by wave and by infection status can be found in the supplementary material (www.sjweh.fi/article/4099, tables S1 and S2). Of note, relatively (but not absolutely) more persons who were fully vaccinated at the start of wave 3 subsequently got infected with SARS-CoV-2 compared to not (fully) vaccinated persons.

**Table 2:** Descriptive characteristics of the total study population (N=2 021 309) stratified by overall job exposure matrix (JEM) score.^a^

Characteristics		Overall JEM score										
		0		1–8		9–12		13–16		17–20		21–24
		N (%)		N (%)		N (%)		N (%)		N (%)		N (%)
Total		368 490 (100.0)		180 204 (100.0)		545 268 (100.0)		524 998 (100.0)		383 655 (100.0)		18 694 (100.0)
Infected		38 808 (10.5)		18 447 (10.2)		50 901 (9.3)		64 002 (12.2)		50 015 (13.0)		3 036 (16.2)
Sex												
	Male	166 840 (45.3)		122 758 (68.1)		382 376 (70.1)		237 014 (45.1)		133 777 (34.9)		10 992 (58.8)
	Female	201 650 (54.7)		57 446 (31.9)		162 892 (29.9)		287 984 (54.9)		249 878 (65.1)		7 702 (41.2)
Region ^b^												
	Capital	138 657 (37.6)		68 585 (38.1)		143 649 (26.3)		139 197 (26.5)		100 877 (26.3)		7 641 (40.9)
	Metropolitan	48 321 (13.1)		25 118 (13.9)		68 352 (12.5)		74 619 (14.2)		51 370 (13.4)		3 093 (16.5)
	Provincial	80 077 (21.7)		38 366 (21.3)		129 271 (23.7)		122 401 (23.3)		91 207 (23.8)		3 545 (19.0)
	Commuter	52 226 (14.2)		25 538 (14.2)		92 354 (16.9)		84 375 (16.1)		60 917 (15.9)		2 091 (11.2)
	Rural	49 208 (13.4)		22 595 (12.5)		111 636 (20.5)		104 395 (19.9)		79 283 (20.7)		2 324 (12.4)
Country of birth												
	Denmark	333 923 (90.6)		163 019 (90.5)		490 967 (90.0)		469 573 (89.4)		306 079 (79.8)		14 184 (75.9)
	EU	13 290 (3.6)		6330 (3.5)		21 825 (4.0)		19 290 (3.7)		19 959 (5.2)		1604 (8.6)
	Western (not EU)	10 038 (2.7)		4936 (2.7)		14 082 (2.6)		14 709 (2.8)		17 833 (4.6)		1110 (5.9)
	Non-western	11 239 (3.1)		5919 (3.3)		18 394 (3.4)		21 426 (4.1)		39 784 (10.4)		1796 (9.6)
Vaccination status												
	Fully ^c^ at the start of Wave 3	21 209 (5.8)		9315 (5.2)		46 978 (8.6)		49 083 (9.3)		53 132 (13.8)		3061 (16.4)
Household income												
	<563 000.00 DKK	94 945 (25.8)		36 967 (20.5)		159 126 (29.2)		178 791 (34.1)		187 976 (49.0)		11 815 (63.2)
	≥563 000.00 DKK	273 545 (74.2)		143 237 (79.5)		386 142 (70.8)		346 207 (65.9)		195 679 (51.0)		6879 (36.8)
ISCO major group												
	0	0 (0.0)		3 996 (2.2)		13 648 (2.5)		0 (0.0)		0 (0.0)		0 (0.0)
	1	28 766 (7.8)		45 456 (25.2)		28 039 (5.1)		0 (0.0)		0 (0.0)		0 (0.0)
	2	83 980 (22.8)		59 644 (33.1)		171 016 (31.4)		236 308 (45.0)		4741 (1.2)		0 (0.0)
	3	91 147 (24.7)		48 371 (26.8)		83 561 (15.3)		33 913 (6.5)		2814 (0.7)		0 (0.0)
	4	164 597 (44.7)		16 386 (9.1)		8404 (1.5)		12 660 (2.4)		0 (0.0)		0 (0.0)
	5	0 (0.0)		6336 (3.5)		1713 (0.3)		102 270 (19.5)		257 716 (67.2)		12 350 (66.1)
	6	0 (0.0)		12 (0.0)		9471 (1.7)		2826 (0.5)		0 (0.0)		0 (0.0)
	7	0 (0.0)		<5 (0.0)		132 029 (24.2)		61 595 (11.7)		0 (0.0)		0 (0.0)
	8	0 (0.0)		0 (0.0)		86 023 (15.8)		17 766 (3.4)		10 996 (2.9)		6344 (33.9)
	9	0 (0.0)		0 (0.0)		11 364 (2.1)		57 660 (11.0)		107 388 (28.0)		0 (0.0)

^a^ Numbers are presented as absolute frequencies (relative frequencies). ^b^ Missing values in region: NA <5 for overall JEM score 0, 1 and 4; NA = 6 for overall JEM ccore 2; NA = 11 for overall JEM score 3. ^c^ Fully vaccinated means either one Janssen vaccination or two vaccinations of Moderna, Pfizer-BioNTech or AstraZeneca.

Table 3 shows an overview of the average test frequency for each risk score of each dimension of the COVID-19-JEM, stratified by wave. The table showed the highest risk scores often contained the highest test frequency per person, although these differences were not substantial. The percentage of the population that was infected was also, with few exceptions, observed to be the highest in the group with the highest risk score. Average test frequency per person and percentages of infections for the entire time period (not stratified by wave) can be found in supplementary table S3.

**Table 3:** Number of participants, average test frequency and percentage SARS-CoV-2 infections for all risk scores per dimension in the three waves.

		WAVE 1 26/2/2020–24/8/2020 Tests (N=928 648)				WAVE 2 25/8/2020–21/6/2021 Tests (N=29 100 357)				WAVE 3 22/6/2021–15/12/2021 Tests (N=13 544 810)		
	Risk score	Population (N)	Average test frequency per person ^a^	Infected ^b^ (%)		Population (N)	Average test frequency per person	Infected (%)		Population (N)	Average test frequency per person	Infected (%)
Number of contacts	0	460 460	0.4	0.2		459 363	14.4	5.1		435 869	7.5	5.4
	1	618 191	0.4	0.2		616 898	13.0	4.7		587 884	6.4	4.9
	2	562 724	0.6	0.6		559 134	15.2	6.4		523 198	7.0	5.5
	3	379 934	0.5	0.5		378 140	15.9	6.6		353 305	8.0	6.5
Nature of contacts	0	461 086	0.4	0.2		459 987	14.4	5.1		436 445	7.5	5.4
	1	701 735	0.4	0.2		700 147	12.7	4.9		665 971	6.3	4.8
	2	686 817	0.5	0.5		683 675	15.7	6.3		640 723	7.8	6.2
	3	171 671	0.8	1.1		169 726	16.6	7.4		157 117	7.2	5.3
Contaminated												
workspaces	0	461 086	0.4	0.2		459 987	14.4	5.1		436 445	7.5	5.4
	1	706 282	0.4	0.2		704 654	12.9	5.0		669 559	6.4	5.0
	2	147 741	0.4	0.3		147 346	14.5	5.2		139 719	7.1	5.6
	3	706 200	0.6	0.7		701 548	16.0	6.7		654 533	7.7	6.0
Location	0	461 086	0.4	0.2		459 987	14.4	5.1		436 445	7.5	5.4
	1	11 622	0.3	0.1		11 609	10.4	4.8		11 054	5.6	5.1
	2	112 274	0.3	0.2		112 076	11.6	4.7		106 864	5.8	4.9
	3	1 436 327	0.5	0.5		1 429 863	14.7	5.9		1 345 893	7.1	5.6
Social distancing	0	461 086	0.4	0.2		459 987	14.4	5.1		436 445	7.5	5.4
	1	611 096	0.4	0.2		609 660	15.0	5.0		579 236	7.2	5.3
	2	540 797	0.4	0.3		539 307	12.7	5.7		508 308	6.5	5.6
	3	408 330	0.7	0.9		404 581	16.0	7.0		376 267	7.5	5.7
												
Face covering	0	500 923	0.4	0.2		499 715	14.5	5.2		473 957	7.4	5.4
	1	718 428	0.6	0.7		713 687	15.2	6.6		666 874	7.3	5.7
	2	726 401	0.4	0.2		724 773	13.6	4.9		689 273	6.7	5.1
	3	75 557	0.5	0.3		75 360	16.0	6.9		70 152	8.0	7.4
Income insecurity	0	1 548 647	0.5	0.4		1 542 368	14.9	5.4		1 458 570	7.2	5.4
	1	241 353	0.4	0.3		240 574	12.2	5.7		226 948	6.1	5.2
	2	145 143	0.4	0.3		144 773	14.1	6.4		135 550	7.7	6.6
	3	86 166	0.4	0.4		85 820	13.7	7.7		79 188	6.8	6.8
Migrants	0	981 067	0.5	0.4		977 037	15.8	5.5		923 391	7.7	5.5
	1	425 873	0.4	0.2		424 884	12.7	5.0		403 439	6.4	5.1
	2	306 342	0.4	0.4		305 121	13.3	6.1		286 554	7.0	5.8
	3	308 027	0.5	0.5		306 493	13.5	6.4		286 872	6.4	5.5
Overall JEM score	0	368 490	0.4	0.2		367 612	14.7	5.2		348 638	7.6	5.4
	1–8	180 204	0.4	0.2		179 760	14.3	5.0		170 693	7.0	5.2
	9–12	545 268	0.4	0.2		544 112	13.1	4.6		519 032	6.4	4.8
	13–16	524 998	0.5	0.6		522 055	15.5	6.1		490 135	7.5	5.9
	17–20	383 655	0.5	0.6		381 367	14.9	7.0		354 672	7.2	5.9
	21–24	18 694	0.4	0.3		18 629	13.7	8.3		17 086	7.4	8.4

^a^ Amount of tests / population. ^b^ Amount of first positive tests / population ×100.

In the dimensions on transmission risk and mitigation measures, higher risk scores were generally, but not consistently, associated with higher odds for a SARS-CoV-2 infection (table 4). Generally, associations in these dimensions were strongest during wave 1 with significant positive odds ratios (OR) ranging from 1.08 [95% confidence interval (CI) 1.00–1.17] for risk score 1 in social distancing to 5.09 (95% CI 4.72–5.50) for risk score 3 in nature of contacts. Associations between occupational exposure and SARS-CoV-2 infection decreased in wave 2 (ranging from 1.06 (95% CI 1.03–1.09) for risk score 2 in location to 1.60 (95% CI 1.57–1.64) for risk score 3 in nature of contacts). Stratified associations by vaccination status are presented in table 4. Associations between the transmission risk factors and mitigation measures further decreased in wave 3 among both fully and not (fully) vaccinated people, with the lowest odds for the fully vaccinated group. For both precarious work dimensions, income insecurity and migrants associations were weak and, in some of the risk scores, significant opposite associations were found. Significant opposite associations were also found in the overall score groups 1–12 both in wave 2 and among the not (fully) vaccinated group of wave 3. Associations for the entire time period can be found in supplementary table S4.

**Table 4:** Associations ^a^ between COVID-19-JEM dimensions and SARS-CoV-2 infection during the first, second and third wave of the pandemic, Denmark 2020–2021.[OR=odds ratio; CI=confidence interval; Ref=reference]

		WAVE 1 26/2/2020–24/8/2020 (N=2 021 309)		WAVE 2 25/8/2020–21/6/2021 (N=2 013 535)		WAVE 3 22/6/2021–15/12/2021 (N=1 900 256)		
Dimension	Risk score	OR (95% CI) adjusted		OR (95% CI) adjusted		Not (fully) vaccinated (N=1 481 909)		Fully vaccinated (N=418 347)
						OR (95% CI) adjusted		OR (95% CI) adjusted
Number of contacts	0	Ref		Ref		Ref		Ref
	1	1.04 (0.96–1.12)		1.01 (0.99–1.02)		1.00 (0.98–1.02)		1.00 (0.95–1.05)
	2	2.95 (2.75–3.16)		1.39 (1.36–1.41)		1.23 (1.21–1.26)		1.03 (0.98–1.08)
	3	2.16 (2.00–2.33)		1.29 (1.27–1.32)		1.29 (1.26–1.31)		1.08 (1.02–1.13)
Nature of contacts	0	Ref		Ref		Ref		Ref
	1	1.13 (1.05–1.22)		1.07 (1.05–1.08)		1.01 (0.99–1.03)		0.99 (0.94–1.04)
	2	2.04 (1.91–2.19)		1.25 (1.23–1.27)		1.24 (1.21–1.26)		1.09 (1.04–1.14)
	3	5.09 (4.72–5.50)		1.60 (1.57–1.64)		1.45 (1.40–1.51)		0.97 (0.92–1.03)
Contaminated workspaces	0	Ref		Ref		Ref		Ref
	1	1.17 (1.09–1.27)		1.10 (1.08–1.12)		1.05 (1.03–1.07)		1.02 (0.97–1.08)
	2	1.16 (1.03–1.30)		1.01 (0.99–1.04)		1.06 (1.03–1.09)		0.98 (0.92–1.05)
	3	2.94 (2.75–3.14)		1.36 (1.34–1.38)		1.27 (1.25–1.30)		1.04 (0.99–1.09)
Location	0	Ref		Ref		Ref		Ref
	1	0.60 (0.35–1.04)		0.99 (0.90–1.07)		1.02 (0.93–1.11)		0.80 (0.57–1.10)
	2	1.08 (0.93–1.26)		1.06 (1.03–1.09)		1.06 (1.03–1.10)		1.01 (0.92–1.10)
	3	2.05 (1.92–2.18)		1.22 (1.20–1.24)		1.15 (1.13–1.17)		1.03 (0.99–1.08)
Social distancing	0	Ref		Ref		Ref		Ref
	1	1.08 (1.00–1.17)		1.07 (1.05–1.09)		1.10 (1.08–1.12)		1.05 (1.00–1.11)
	2	1.37 (1.27–1.49)		1.21 (1.19–1.23)		1.13 (1.11–1.16)		1.04 (0.98–1.10)
	3	4.28 (3.99–4.60)		1.42 (1.39–1.45)		1.24 (1.22–1.27)		1.00 (0.95–1.05)
Face covering	0	Ref		Ref		Ref		Ref
	1	2.84 (2.66–3.03)		1.28 (1.26–1.30)		1.15 (1.13–1.17)		0.96 (0.92–1.01)
	2	1.12 (1.04–1.21)		1.07 (1.05–1.09)		1.07 (1.05–1.09)		1.04 (0.99–1.09)
	3	1.16 (1.00–1.36)		1.33 (1.29–1.38)		1.39 (1.34–1.44)		1.27 (1.15–1.40)
Income insecurity	0	Ref		Ref		Ref		Ref
	1	0.86 (0.80–0.93)		1.04 (1.02–1.06)		0.95 (0.93–0.97)		1.06 (1.00–1.12)
	2	0.56 (0.51–0.63)		0.98 (0.95–1.00)		1.03 (1.01–1.06)		1.09 (1.01–1.18)
	3	0.69 (0.61–0.77)		1.08 (1.05–1.11)		1.02 (0.99–1.06)		0.98 (0.90–1.08)
Migrants	0	Ref		Ref		Ref		Ref
	1	0.65 (0.60–0.70)		0.93 (0.92–0.95)		0.92 (0.90–0.94)		0.97 (0.92–1.02)
	2	1.05 (0.98–1.13)		1.04 (1.02–1.06)		1.00 (0.98–1.02)		1.01 (0.96–1.06)
	3	1.20 (1.12–1.27)		1.12 (1.10–1.14)		1.03 (1.01–1.06)		0.95 (0.91–0.99)
Overall JEM score	0	Ref		Ref		Ref		Ref
	1–8	1.06 (0.95–1.19)		0.97 (0.95–1.00)		0.96 (0.94–0.99)		0.97 (0.90–1.04)
	9–12	1.05 (0.96–1.15)		0.97 (0.95–0.99)		0.96 (0.94–0.98)		0.93 (0.88–0.98)
	13–16	2.65 (2.46–2.86)		1.29 (1.26–1.31)		1.27 (1.24–1.29)		1.04 (0.99–1.10)
	17–20	2.61 (2.40–2.83)		1.32 (1.30–1.35)		1.17 (1.14–1.20)		0.99 (0.93–1.04)
	21–24	1.51 (1.17–1.94)		1.32 (1.25–1.39)		1.31 (1.23–1.39)		1.25 (1.03–1.50)

^a^ Adjusted for sex, age, region, country of birth and pre-tax household income.

After repeating the analyzes using a test-negative design, the risk-estimates barely changed (supplementary table S5). When dividing the population of the normal analyzes into ‘inhabitants of the capital region and ‘inhabitants of the other regions’, associations appeared to be much stronger for the capital region, where the relative test positive rate is higher (supplementary table S6).

## Discussion

Overall, higher risk scores of the six dimensions on transmission risk and mitigation measures were associated with higher odds for SARS-CoV-2 infection in all three waves, but magnitude of associations gradually decreased over time. The finding that, in the first six dimensions, higher risk scores were associated with higher infection rates are in line with the validation of both the Dutch and the UK version of the COVID-19-JEM (20, 21). The dimension ‘nature of contacts’ showed the strongest associations with SARS-CoV-2 infection in all three waves together with the dimension ‘social distancing’ in waves 1 and 2. These results are in line with a previous study, which also identified exposure to infected persons and physical proximity as important risk factors for SARS-CoV-2 infection, explaining most of the occupational variance in COVID-19 prevalence (8). The two dimensions on precarious work were in general not associated with a higher risk of SARS-CoV-2 infection and showed sometimes even an inverse association.

In line with the UK validation study (20), the associations between risk scores and SARS-CoV-2 infection decreased during the pandemic, with the lowest OR in wave 3. These findings are not surprising because the COVID-19-JEM was developed in the context of strict government measures, which was the case during the first wave. In the second and third wave, the more relaxed legislative restrictions such as the reopening of schools and other public spaces may have resulted in much higher transmission rates in the society in general, which in turn may have decreased the relative importance of work-related transmission.

The weak and even inverse associations in the precarious work dimensions are not perfectly in line with the literature (28–33). Potentially precarious workers were less likely to seek testing; for example due to fear of consequences of a positive test result such as not being able to hold a job. Literature on disparities in test frequencies is inconsistent (33), but it is noteworthy that Denmark implied a mass testing policy, making testing freely and abundantly available and thereby taking away many practical testing barriers. Importantly, the COVID-19-JEM does not intend to assess individual risk factors but occupational groups risk factors, where the majority of the workers would still be non-precarious workers. The JEM assumed that for jobs where there were more migrant workers, restriction would not be implemented as strictly as for other jobs or not be followed as carefully, which would result in higher risk of transmission within this occupation.

The COVID-19-JEM was developed early in the pandemic, whereas currently much more knowledge exists about SARS-CoV-2 transmission, and new variants have influenced the transmission risk (34). Although the COVID-19-JEM does show to be useful in assessing occupational risk on SARS-CoV-2 exposure, it is recommended to revise the JEM based on the current state of knowledge. Based on the findings of this study and the studies of the UK and The Netherlands (20, 21), it is recommended to revise or exclude the precarious work dimensions. Moreover, ventilation could be considered as an important dimension of risk as well (35, 36) since airborne transmission is now considered to be a major transmission route of SARS-CoV-2 (34). Although work location (working in- versus outdoors) is a useful starting point, it is recommended to refine this dimension by including ventilation aspects. However, very little data are available on ventilation rates in occupational settings, and these will be highly variable across workplaces and days, hence impossible to provide useful assessments at a job level. Furthermore, the proportion of fully vaccinated people might also be of interest as a mitigation measure. Finally, with the changing legislative restrictions, work patterns such as working from home have changed since the development of the COVID-19-JEM in 2020. Therefore, researchers should be aware of the governmental measures during their study period and might consider to adjust the COVID-19-JEM scores as suggested by Oude Hengel et al (18).

A major strength of this study is the access to the nationwide database of SARS-CoV-2 test results among all workers, allowing for almost all occupations to be represented. This made it possible to investigate the associations between the COVID-19-JEM and SARS-CoV-2 infection in the entire working population and across different waves of the pandemic. Government measures, new upcoming variants of SARS-CoV-2 and vaccination status changed during the pandemic, thus it was not surprising that the associations between the COVID-19-JEM and SARS-CoV-2 infection decreased over time. One major limitation of the current study is that the most recent occupational data available was gathered at the end of 2019. Since then, people might have started or stopped working, while others may have changed jobs and new jobs have been created during the pandemic (eg, working in COVID-19 test centers). Therefore, some misclassification will exist with regards to occupation and the corresponding risk scores of the COVID-19-JEM. Moreover, potential intra-occupational variability due to, for example, differences in institutional COVID-19 measures might have caused misclassification with regards to risk scores as well. Another limitation is that misclassification could also have occurred for infection status. No data were available of rapid antigen tests taken at home and tests taken by some commercial test centers. Moreover, some of the test results might in fact have been false positive or false negative tests, causing people to be misclassified for SARS-CoV-2 infection. However, this misclassification might be limited as the majority of the positive tests were PCR tests, with high sensitivity and specificity (37). However, it should be noted that 23.7% of the positive tests were rapid antigen test, which do have a reasonable specificity but have a significantly lower sensitivity than rt-PCR tests resulting in an increased risk of primarily false-negative test outcomes (38–41). Finally, no data were available to differentiate between SARS-CoV-2 infections resulting from exposure at work versus outside work. The literature shows that exposure outside of the workplace is a highly important risk factor for SARS-CoV-2 infection (10, 42–47). However, we expect a relatively equal distribution of exposure at home across risk groups, and we do not expect that exposure at home influenced our results to a large extent.

This study has shown that the COVID-19-JEM seems to be a promising tool to assess occupational risk of exposure to SARS-CoV-2 in Denmark and other countries (20, 21). However, the efficiency depends on applied legislative restrictions and the vaccination status in the population of interest. The COVID-19-JEM was designed for a specific set of government measures and the attenuating associations between its dimensions and positive SARS-CoV-2 tests show that it is important to adjust the occupational risk assessment for all dimensions to the changing state of the pandemic. Moreover, this study has shown that working from home and the availability of preventive measures at work such as wearing face coverings and the ability to keep social distance are associated with a lower risk of SARS-CoV-2 infection. It will not be possible for some occupations to implement all of these preventive measures, for example taking care of COVID-19 patients necessarily brings you in close proximity to high risk contacts. However, this study does suggest that preventive measures have been successful and should be applied where possible.

The COVID-19-JEM can be useful for research investigating the role of occupational exposure across different jobs over time as well as for research investigating the role of the worksite in transmission by comparing a SARS-CoV-2 exposure setting to other settings (eg, household composition, residential area). Moreover, the JEM might contribute to insights about the occupational spread of other airborne infectious agents that mainly spread between people who are in close contact with each other.

### Practical implications

The internationally established COVID-19-JEM is a promising tool in the Danish context to assess occupational exposure to SARS-CoV-2 and potentially other airborne infectious agents. However, it should be adjusted to changing government measures, and its performance depends on the vaccination status of the population of interest.

### Ethics approval

Permissions to retrieve and analyze pseudonymized data through Statistics Denmark were obtained from the Knowledge Center on Data Protection Compliance under the records of processing regarding health science research projects within the Capitol Region of Denmark (P-2020-897), Statistics Denmark (P-708121) and the National Board of Health Data (FSEID-00005368).

Ethical approval is by Danish law not required for studies that are entirely based on public registries.

## Supplementary material

Supplementary material
